# Ecthyma Gangrenosum and Pyelonephritis in an Older Patient with Controlled Diabetes but without HIV or Malignancy

**DOI:** 10.59468/2836-8525/128

**Published:** 2025-09-10

**Authors:** Shuhua Guo, Minghsun Liu

**Affiliations:** 1 Southern California Hospital Culver City, California; 2 Coast Plaza Hospital, Norwalk, California; 3 Infectious Diseases, Centinela Hospital Medical Center/Prime West Internal Medicine Program, Inglewood, California; 4 Woundtech, Hollywood, Florida

**Keywords:** Ecthyma gangrenosum, dermatological manifestation, septic shock

## Abstract

Ecthyma gangrenosum is a rare dermatological manifestation often linked to Pseudomonas aeruginosa bacteremia, typically occurring in immunocompromised individuals. We present a case of ecthyma gangrenosum in a 69-year-old male with diabetes mellitus, but without HIV, malignancy, or chronic immunosuppressive therapy. The patient exhibited septic shock, pyelonephritis, and pneumonia, and was eventually diagnosed with ecthyma gangrenosum due to systemic Pseudomonas aeruginosa infection. This case highlights the occurrence of ecthyma gangrenosum in an older patient with metabolic comorbidities and age-related immune dysfunction, but without classic immunosuppressive conditions. It underscores the importance of early recognition and intervention in such populations, and the need for a nuanced approach to immune status in elderly patients with diabetes.

## Introduction:

Ecthyma gangrenosum is most associated with Pseudomonas aeruginosa bacteremia in immunocompromised hosts. However, it can rarely occur in patients without overt immunosuppression. Older adults with diabetes may have subtle or acquired immune dysfunction, even in the absence of HIV or malignancy (1). Here, we report a case in an elderly patient with diabetes and subacute malnutrition, emphasizing the need for vigilance even in the absence of classic risk factors.

## Case Presentation

A 69-year-old male with a history of diabetes mellitus, hypertension, and hyperlipidemia presented to the emergency department following a near-syncopal episode witnessed by neighbors. He reported having loose black stools for the past week and anuria for several days. On arrival, he was hypothermic with a temperature of 94.4°F, borderline hypotensive, and tachycardic with a heart rate in the 100s. His oxygen saturation was 98% on room air. Physical examination revealed multiple cutaneous lesions, particularly on the left hip and sacrum, which were painless and appeared as circular, erythematous macules with central necrosis.

The patient was not bedbound prior to admission but had experienced progressive functional decline and reduced oral intake over several weeks, resulting in mild hypoalbuminemia and evidence of malnutrition. He had not been recently hospitalized and lived independently until the week prior to presentation.

Initial laboratory workup revealed significant leukocytosis (WBC 47.7 × 10^3/µL), hemoglobin 13.6 g/dL, sodium 124 mEq/L, potassium 6.4 mEq/L, CO_₂_ 8 mEq/L, anion gap 38, BUN 261 mg/dL, creatinine 20.13 mg/dL, and elevated creatine kinase (CK 1800 U/L).

Urinalysis showed 10–20 WBCs per high-power field. A non-contrast CT of the abdomen revealed a right lower lobe opacity, moderate bilateral hydronephrosis with extensive left perinephric stranding and edema, and significant prostate enlargement.

After placement of a urinary catheter, 4 liters of urine were drained. Blood, urine, wound, and sputum cultures were obtained, and the patient was admitted to the ICU for septic shock secondary to left pyelonephritis and pneumonia with acute renal failure. The patient was started on empiric antibiotics with vancomycin and ceftriaxone, norepinephrine for hemodynamic support, and hemodialysis. Infectious disease consultation was sought for antibiotic management.

Vancomycin was tapered to doxycycline due to low suspicion of MRSA and the patient’s acute renal failure. On examination, diffuse erythematous macules with central necrosis were noted on his extremities, trunk, and left hip. Ceftriaxone was escalated to cefepime, and later to meropenem and linezolid to broaden antimicrobial coverage for Pseudomonas and Enterococcus. Despite persistent leukocytosis in the 30s for 7 days, his white blood cell counts eventually normalized within 10 days.

Cultures from blood and sputum were positive for P. aeruginosa, sensitive to cefepime. The urine culture was negative, while a wound culture from the left hip also grew P. aeruginosa. As the patient stabilized, linezolid was discontinued, and meropenem was de-escalated to cefepime. The patient’s lesions evolved from erythematous necrotic plaques into ulcerations with black eschar and surrounding erythema, consistent with ecthyma gangrenosum in the healing phase. An HIV antibody test was negative. The patient was treated with a 14-day course of cefepime.

**Figure 1:**
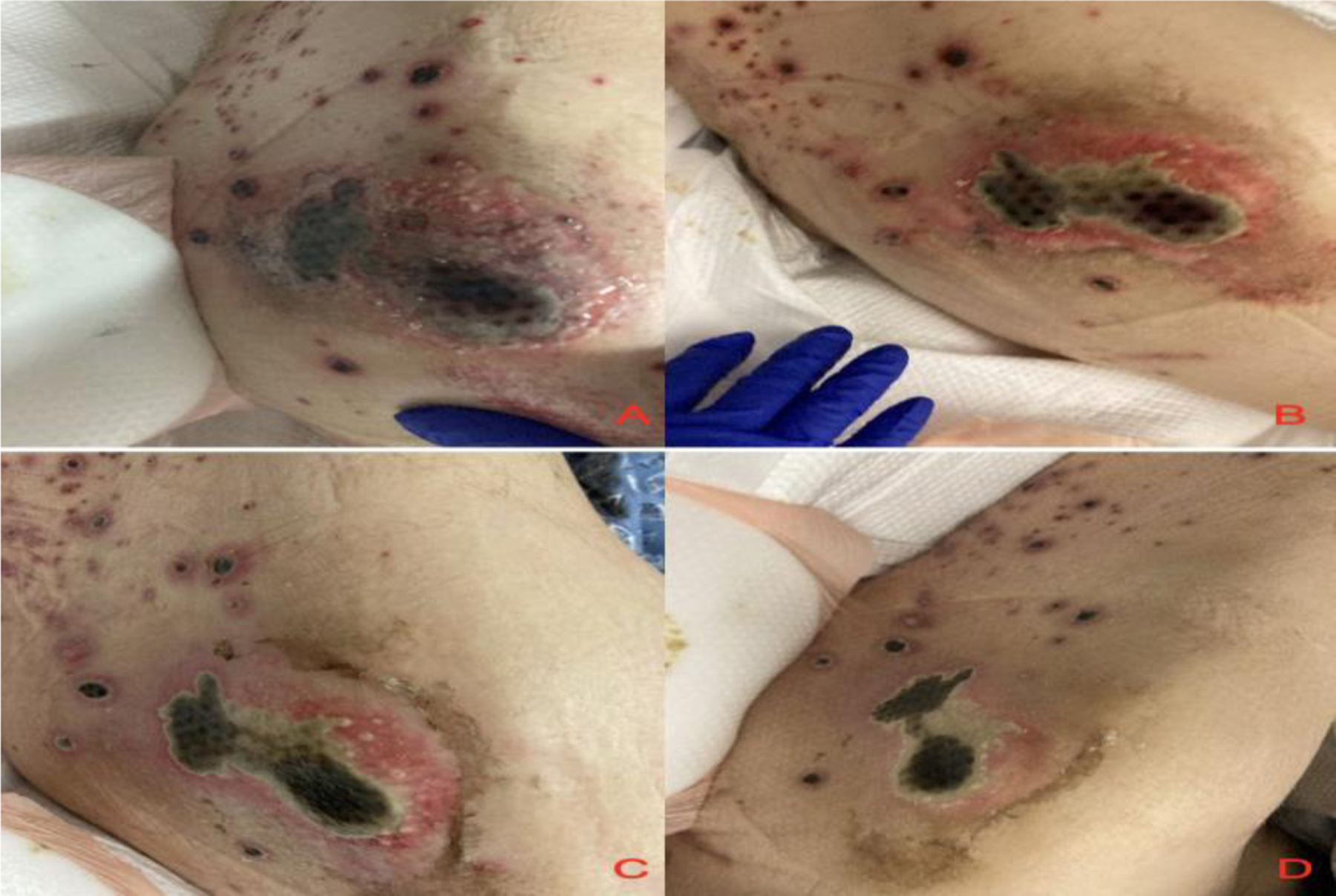
Evolution of ecthyma gangrenosum lesion on the thigh: A) On presentation Hospital Day 1, B) Hospital Day 2 , C) Hospital Day 7, D) Hospital Day 10Although there was significant interval improvement in the ecthyma gangrenosum lesions, complete resolution was not achieved by the time of discharge (Figure 1D). The patient was transferred to a short-term care facility to complete his antimicrobial treatment.

A consolidated timeline correlating clinical events, laboratory findings, and lesion evolution is provided in Table 1. Figure 1 demonstrates the progression of a representative lesion on the left thigh from presentation through day 10.

## Discussion

Ecthyma gangrenosum results from bacterial invasion of blood vessels, leading to ischemic necrosis, and is most associated with P. aeruginosa infection. This condition typically occurs in immunocompromised patients, such as those with neutropenia, malignancy, or HIV. P. aeruginosa produces several toxins that promote tissue breakdown (2), and ecthyma gangrenosum has been documented in fewer than 3% of P. aeruginosa bacteremia cases (3). Other pathogens that can cause ecthyma gangrenosum-like presentations include MRSA, Streptococcus pyogenes, Escherichia coli, and various viruses such as herpes simplex (4).

This patient’s presentation of leukocytosis, tachycardia, hypotension, and elevated lactate was consistent with septic shock. Cultures from blood, sputum, and the left hip wound were positive for P. aeruginosa, supporting the diagnosis of a systemic infection. The exact source of bacteremia remains unclear. In classic ecthyma gangrenosum, the skin lesions result from hematogenous seeding during bacteremia, rather than the skin serving as the primary source. In this case, both blood and wound cultures were positive for P. aeruginosa, while the urine culture was negative. Although the patient had skin breakdown, it is most consistent with the established pathophysiology that bacteremia, potentially originating from a urinary, respiratory, or gastrointestinal source, led to secondary cutaneous seeding. The possibility of direct inoculation through a skin breach cannot be entirely excluded but is less likely given the clinical context and distribution of lesions.

While the patient did not have classic immunosuppressive conditions such as malignancy, HIV, or chronic corticosteroid use (5,6), his advanced age, diabetes, and subacute malnutrition likely contributed to impaired immune function.

Several case reports have documented ecthyma gangrenosum in pediatric and adult patients without classic immunosuppression (3, 7–12), but most lacked bacteremia or severe systemic illness. Our case is novel in demonstrating severe, widespread ecthyma gangrenosum with bacteremia in an elderly patient with diabetes and malnutrition, but without HIV, malignancy, or recent immunosuppressive therapy. This highlights the need to consider ecthyma gangrenosum in elderly or metabolically compromised patients, even in the absence of traditional risk factors.

## Conclusion

This case illustrates that ecthyma gangrenosum may develop in older patients with diabetes and subacute malnutrition, even in the absence of classic immunosuppressive conditions, emphasizing the need for early recognition and prompt initiation of antimicrobial therapy to optimize outcomes.

## Figures and Tables

**Table 1:** Consolidated Timeline of Clinical Events and Lesion Evolution

Day	Event
1	Presentation: near-syncope, loose black stools, anuria, hypothermia, hypotension, tachycardia, multiple painless cutaneous lesions (ecthyma gangrenosum) on left hip, sacrum, trunk, and extremities. Labs: leukocytosis, acute renal failure. Imaging: pneumonia, bilateral hydronephrosis, perinephric stranding.
2–3	Antibiotic adjustment: vancomycin to doxycycline, ceftriaxone to cefepime, then meropenem and linezolid.
4–10	Persistent leukocytosis, positive blood and wound cultures for *P. aeruginosa*, urine culture negative. Lesions evolve to maroon macules with central necrosis. Patient switched back to cefepime.
11–14	Stabilization, WBC normalizes, HIV negative, continued cefepime.
15	Discharge to short-term care, significant improvement in lesions but not fully resolved.
